# The search for gastrointestinal inflammation in autism: a systematic review and meta-analysis of non-invasive gastrointestinal markers

**DOI:** 10.1186/s13229-023-00575-0

**Published:** 2024-01-17

**Authors:** Nisha E. Mathew, Delyse McCaffrey, Adam K. Walker, Kylie-Ann Mallitt, Anne Masi, Margaret J. Morris, Chee Y. Ooi

**Affiliations:** 1 School of Clinical Medicine, Discipline of Paediatrics and Child Health, UNSW Medicine and Health, University of New South Wales, Sydney, 2052 Australia; 2 Laboratory of ImmunoPsychiatry, Neuroscience Research Australia, Randwick, NSW 2031 Australia; 3 School of Clinical Medicine, Discipline of Psychiatry and Mental Health, UNSW Medicine and Health, University of New South Wales, Sydney, 2052 Australia; 4 Drug Discovery Biology Theme, Monash Institute of Pharmaceutical Sciences, Monash University, Parkville, VIC 3800 Australia; 5 Sydney School of Public Health, Faculty of Medicine and Health, University of Sydney, Camperdown, NSW 2006 Australia; 6 School of Biomedical Sciences, University of New South Wales, Sydney, 2052 Australia; 7 Department of Gastroenterology, Sydney Children’s Hospital, High Street, Randwick, NSW 2031 Australia

**Keywords:** Autism, Inflammation, Gastrointestinal, Calprotectin, Lactoferrin, Biomarkers

## Abstract

**Background:**

Gastrointestinal symptoms and inflammatory gastrointestinal diseases exist at higher rates in the autistic population. It is not clear however whether autism is associated with elevated gastrointestinal inflammation as studies examining non-invasive faecal biomarkers report conflicting findings. To understand the research landscape and identify gaps, we performed a systematic review and meta-analysis of studies measuring non-invasive markers of gastrointestinal inflammation in autistic and non-autistic samples. Our examination focused on faecal biomarkers as sampling is non-invasive and these markers are a direct reflection of inflammatory processes in the gastrointestinal tract.

**Methods:**

We extracted data from case–control studies examining faecal markers of gastrointestinal inflammation. We searched PubMed, Embase, Cochrane CENTRAL, CINAHL, PsycINFO, Web of Science Core Collection and Epistemonikos and forward and backwards citations of included studies published up to April 14, 2023 (PROSPERO CRD42022369279).

**Results:**

There were few studies examining faecal markers of gastrointestinal inflammation in the autistic population, and many established markers have not been studied. Meta-analyses of studies examining calprotectin (*n* = 9) and lactoferrin (*n* = 3) were carried out. A total of 508 autistic children and adolescents and 397 non-autistic children and adolescents were included in the meta-analysis of calprotectin studies which found no significant group differences (ROM: 1.30 [0.91, 1.86]). Estimated differences in calprotectin were lower in studies with siblings and studies which did not exclude non-autistic controls with gastrointestinal symptoms. A total of 139 autistic participants and 75 non-autistic controls were included in the meta-analysis of lactoferrin studies which found no significant group differences (ROM: 1.27 [0.79, 2.04]).

**Limitations:**

All studies included in this systematic review and meta-analysis examined children and adolescents. Many studies included non-autistic controls with gastrointestinal symptoms which limit the validity of their findings. The majority of studies of gastrointestinal inflammation focused on children under 12 with few studies including adolescent participants. Most studies that included participants aged four or under did not account for the impact of age on calprotectin levels. Future studies should screen for relevant confounders, include larger samples and explore gastrointestinal inflammation in autistic adolescents and adults.

**Conclusions:**

There is no evidence to suggest higher levels of gastrointestinal inflammation as measured by calprotectin and lactoferrin are present in autistic children and adolescents at the population level. Preliminary evidence suggests however that higher calprotectin levels may be present in a subset of autistic participants, who may be clinically characterised by more severe gastrointestinal symptoms and higher levels of autistic traits.

**Supplementary Information:**

The online version contains supplementary material available at 10.1186/s13229-023-00575-0.

## Introduction

Autism is a neurodevelopmental condition associated with increased rates of co-occurring medical conditions including gastrointestinal (GI) disorders [1]. In addition, several studies report higher rates of GI symptoms in paediatric [2, 3] and adult autistic populations [4]. Higher GI symptom rates in autism likely stem from a combination of multifactorial causes, involving genetic, environmental, and behavioural factors. Several gene mutations linked to autism are associated with GI symptoms including impaired motility, constipation and gastro-oesophageal reflux (e.g. CHD8, NOS1, FOXP1 and TCF4) [5]. Autistic traits, such as high levels of restricted and repetitive behaviours and interests, are also associated with less diverse diets in some autistic people [6], which could exacerbate GI issues and contribute to microbial dysbiosis [7]. GI issues interact with other conditions and may worsen sleep problems and increase rates of self-injurious and aggressive behaviours, particularly among non-verbal autistic children [8–10]. Higher rates of internalising symptoms, including anxiety and social withdrawal, which present at elevated rates in the autistic population, have a bidirectional relationship with GI problems such as constipation, diarrhoea, nausea, and stomach pain [11].

There has been little research examining whether these higher rates of GI symptoms are associated with dysregulated immune responses within the GI tract, prompting investigations into whether low-grade GI inflammation is present in the autistic population [12]. Epidemiological evidence from a recent meta-analysis suggests that autistic people are more likely to be diagnosed with inflammatory bowel disease (IBD) than non-autistic people highlighting the need to screen for GI inflammation in the autistic population [13]. Comparisons of endoscopic findings have been limited to small sample sizes and have found conflicting evidence as to whether GI inflammation is present in the autistic population [3, 14]. While endoscopy remains the gold standard to identify GI inflammation, inflammatory biomarkers measured in faecal samples are often used in research and clinical settings as non-invasive markers of GI inflammation to avoid the risks of endoscopy and general anaesthesia [15, 16]. The direct contact with mucosa of the GI tract makes faecal biomarkers a more direct, non-invasive marker of intestinal inflammation than plasma or serum biomarkers which could be elevated by non-GI causes of inflammation [17].

Biomarkers of interest include calprotectin and S100 calcium binding protein A12 (S100A12) which are released by neutrophils, monocytes and infiltrating macrophages in response to inflammation in the GI tract [17–19]. Other markers of interest include lactoferrin and secretory Immunoglobulin A (IgA) which are anti-inflammatory glycoproteins secreted by macrophages [15, 18]. Faecal measurements of the dimeric M2-isoform of pyruvate kinase (M2-PK), which is associated with increased cell turnover in the GI tract, is also used as a marker of GI inflammation [15, 19, 20]. Lysozymes are antimicrobial enzymes produced by neutrophils and neopterin which is released by activated T-lymphocytes, and macrophages have also been found to be upregulated in IBD [19]. Alpha1-antitrypsin (AAT), a serine protease inhibitor produced by a range of cells including: hepatocytes, neutrophils, monocytes-macrophages, enterocytes, and Paneth cells, and polymorphonuclear neutrophil elastase (PMN-E), a serine protease produced by neutrophils, are both upregulated in response to GI inflammation [19]. GI symptoms in autism have been explored in relation to the microbiome [21]; however, evidence of GI abnormalities as reflected in faecal biomarkers of inflammation has been conflicting. Given higher rates of GI symptoms in the autistic population and concerns of higher rates of inflammation-driven GI conditions, this paper sought to conduct a systematic review of markers of faecal markers of GI inflammation.

## Materials and methods

The systematic review and meta-analysis were undertaken and reported in accordance with the Preferred Reporting Items for Systematic Reviews and Meta-Analysis (PRISMA) statement [22] and was pre-registered on PROSPERO (CRD42022369279).

### Literature search

We searched PubMed, Embase, Cochrane Central Register of Controlled Trials (CENTRAL), CINAHL, PsycINFO, Web of Science Core Collection and Epistemonikos using strategies developed for each database (Additional file 1). Scopus and Web of Science were used to examine backwards and forwards citations of all studies included in this review.

### Selection of studies

The titles and abstracts of all studies retrieved by the search were reviewed by two researchers (NM and DM). The full texts of relevant studies were then screened by NM to identify eligible studies that met the inclusion and exclusion criteria. The criteria for inclusion were (1) the measurement of inflammatory biomarkers in faeces (2) autism diagnoses confirmed by standardised diagnostic tools or by a medical professional in line with the criteria outlined in the DSM-III, IV or 5 or ICD-10 or 11. The exclusion criteria were (1) studies of biopsies and endoscopies, as the focus of this review is on biospecimens collected using minimally invasive sampling procedures, (2) studies that did not provide data as absolute concentrations (e.g. relative data reported by Western blots) or insufficient information regarding the method of data quantification, (3) studies that only report data from analyses of the microbiome, as the focus of this meta-analysis is on markers of GI inflammation.

### Data extraction and risk of bias

The means and standard deviations of the faecal biomarker levels in autistic and non-autistic cohorts were extracted if available. If means and standard deviations were not reported, they were derived from sample size, median, IQR, minimum, or maximum values [23]. Risk of bias in all included studies was assessed using a study specific adaptation of the Newcastle–Ottawa Scale (NOS) for case–control studies carried out by two researchers (NM and DM) (Additional file 1). We extracted the following data from all included studies: the age and sex of participants, measurement of concurrent psychiatric or medical conditions, country of publication and publication year, details of recruitment settings, any variables used to match participants, method of faecal biomarker quantification. As considerable interassay variability has been reported between different commercial calprotectin assays [24–26], details of the assays used by individual studies were also extracted. To examine the generalisability of the literature, the inclusion of autistic participants with limited verbal and/or cognitive abilities was also coded based on reports of communication ability, adaptive functioning or cognitive ability in line with the criteria developed by Stedman et al. [27].

### Statistical analysis

Meta-analyses were performed when at least two studies that could be combined were identified. Random-effects meta-analyses were conducted in R 4.2.1 (R Foundation for Statistical Computing, Vienna, Austria) using the *metafor* package. To control for the substantial variability in biomarker concentrations between laboratories and assays, the ratio of mean (RoM) faecal biomarker levels in autistic and non-autistic cohorts and standard errors were generated for each comparison, log transformed and pooled for meta-analysis [28]. Standardised mean differences were also calculated and pooled for all analyses. All major findings remained consistent with the analysis of RoM (Additional file 2: Figs. S1, S2). When multiple autistic cohorts were present within a single study, autistic cohorts were combined for the main analysis. When multiple control cohorts were present, individual ratios were generated. Heterogeneity was assessed using *I*^2^ [29].

## Results

### Systemic review of faecal biomarkers of inflammation

**Fig. 1:**
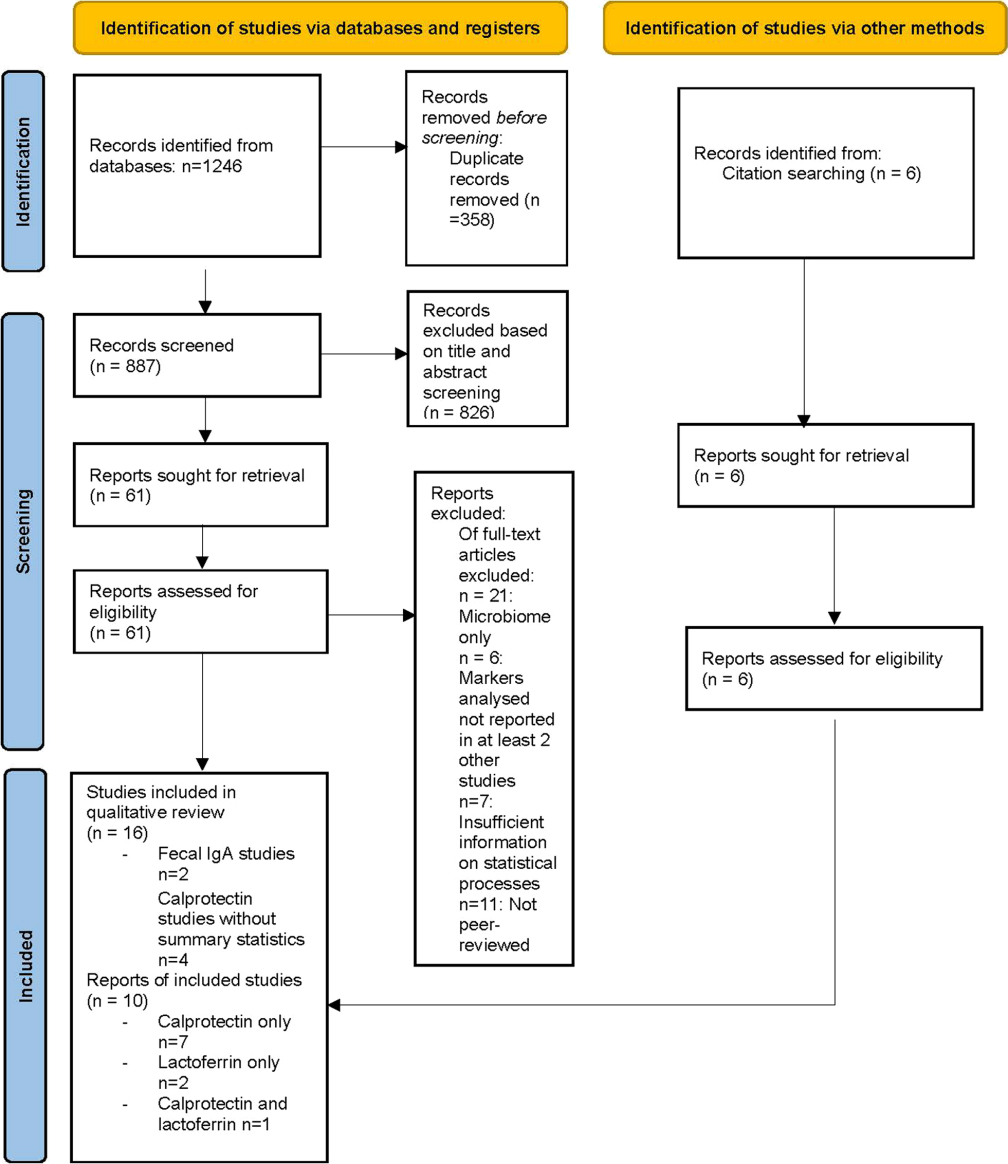
PRISMA flow diagram of search for faecal biomarkers in autism

Many inflammatory markers used in studies of GI inflammation (e.g. M2-PK, S100A12, AAT) have not been examined in the autistic population. Single studies found lower faecal levels of lysozyme, particularly among those with higher probiotic usage [30], cortisol and glutamate metabolites [31] and comparable levels of PMN-E [30] among autistic participants relative to controls. A small Swedish study found that elevated rectal nitric oxide levels, defined as levels above < 250 parts per billion, were reported in 27% of surveyed autistic participants (6/22) and in 9% of surveyed controls (2/22) [32] (Fig. 1).

Two studies examined the levels of secretory IgA in faecal samples, but the results of these studies could not be synthesised as one study reported results as optical densities rather than concentrations. Adams et al. found comparable levels of faecal IgA concentrations in 39 control children and adolescents and 58 autistic children and adolescents, (55 participants with a DSM-IV diagnosis of Autistic disorder and 3 with a diagnosis of Asperger’s disorder) [30]. Zhou et al. [33] found higher levels of faecal IgA, reported as optical densities, in the stools of 43 autistic participants relative to 31 gender and age matched controls. Subsequent analysis of the IgA data found IgA levels could not distinguish between autistic children and adolescents with GI problems and those without however abnormal IgA levels were associated with altered microbiome composition [34].

There were sufficient studies reporting summary statistics to enable a meta-analysis of two faecal inflammatory markers: calprotectin and lactoferrin.

#### Calprotectin

Of the 12 peer-reviewed studies examining stool calprotectin in autism, one study did not include comparisons with a control group [35] and three studies could not be included in the meta-analysis as relevant summary statistics could not be retrieved [36–38]. Median calprotectin levels of 77.12 μg/g (IGR = 135.69 μg/g) were found in a study of 80 autistic preschoolers participating in a trial of probiotic supplementation [35]. This study found that calprotectin levels were not significantly different between preschoolers with and without GI symptoms. In the remaining three studies, no differences were found between levels of calprotectin in autistic children and adolescents relative to non-autistic controls [37, 38], siblings or first-degree relatives [36, 37].

**Fig. 2:**
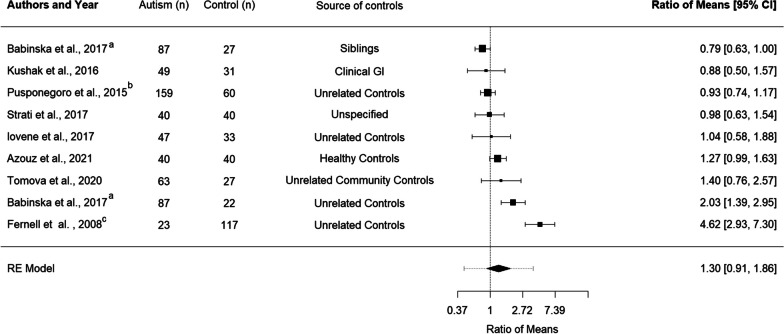
Forest plot indicating mean levels of calprotectin in autistic children and adolescents relative to control participants. **A** Babinská, et al. [40] reported comparisons between calprotectin in autistic participants and calprotectin in siblings and unrelated controls. Individual comparison ratios were generated for both groups. **B** The two autistic groups in Pusponegoro et al. were combined for analysis **C** Calprotectin data were extracted from the supplementary materials of Fernell et al. [32] and the control group data were published in Fagerberg et al. [45]

Eight studies, with nine comparison groups and a total of 508 autistic children and adolescents and 397 control children and adolescents, were included in the meta-analysis of calprotectin [14, 32, 39–45]. The mild-moderate and severe autism groups included in Pusponegoro et al. [42] were combined to generate a single ROM for analysis. Two ROM were generated for Babinská et al.’s study as this study included two control groups, one consisting of non-autistic siblings and the other of unrelated controls [44]. Overall, this meta-analysis found no significant difference between the levels of calprotectin in autistic children and adolescents relative to comparison groups (ROM 1.30 [0.91, 1.86], *p* = 0.178) (Fig. 2).

Significant heterogeneity was found between included studies *I*^2^ = 89.07%. We could not examine the impact of age on the heterogeneity of studies examining calprotectin levels as there was extensive overlap between the average age of participants in this meta-analysis, with few studies including adolescents and the reported demographics suggesting that the majority of participants studied to date were children aged between 5–8 years. Studies that recruited a wide age range did not conduct separate analyses of calprotectin levels in toddlers, children and adolescents [14, 43] and no studies included adult participants. Sensitivity analysis of gender was not possible as most studies used mixed samples [14, 32, 39–43], and the majority of included participants were male (Additional file 3).

Another potential source of heterogeneity is the type of control group (siblings vs. non-siblings or studies which explicitly excluded controls with GI symptoms vs those without GI screening). Only one included study examined calprotectin levels in siblings [40]. This study found higher levels of calprotectin in autistic participants relative to unrelated controls but no differences in comparison with non-autistic siblings [40]. Excluding the sibling comparison in Babinská et al.’s study [40] did not alter the findings but increased the RoM and decreased the variance (Additional file 2: Fig. S3). Similar findings of no differences in calprotectin levels when autistic participants were compared to their non-autistic siblings were reported by two other studies that were not included in this meta-analysis as summary statistics could not be retrieved [36, 37]. Three studies reported the inclusion of control participants with GI symptoms [14, 42, 43]. Lower effect estimates were reported in these three studies than in the other five non-sibling comparisons included in the meta-analysis [32, 39–41, 44].

#### Lactoferrin

**Fig. 3.**
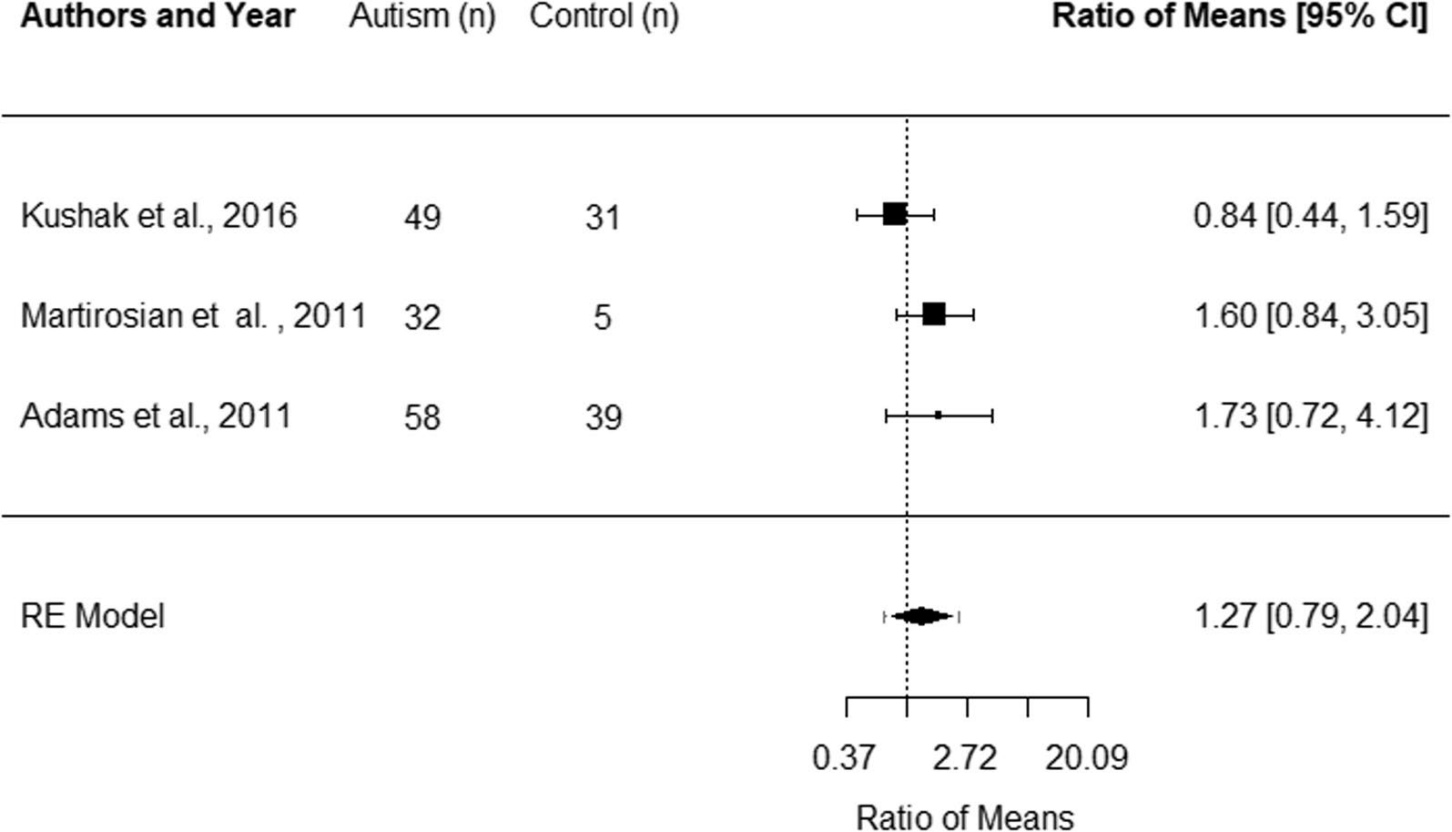
Forest plot of studies comparing lactoferrin levels in autistic children and adolescents relative to control participants

Three studies, with a total of 139 autistic participants and 75 non-autistic controls, were included in the meta-analysis of studies examining lactoferrin levels [14, 30, 46]. There was no significant difference in the lactoferrin levels of autistic children and adolescents relative to controls (ROM: 1.27 [0.79, 2.04]). Heterogeneity was not significant in the meta-analysis of lactoferrin, *I*^2^ = 25.74% (Fig. 3).

### Risk of bias assessment

A significant issue in many studies included in this meta-analysis is the failure to screen control groups. Few studies reported the details of lab blinding and failed to match controls and autistic participants on age and other demographic characteristics such as sex or socioeconomic status (Table 1). Studies that reported matching controls and autistcic children and adolescents often did not detail how participants were matched and often did not account for the variables being matched for in statistical analyses (e.g. paired t tests or regression models controlling for age and sex). There was insufficient information to determine whether autistic participants with limited verbal and/or cognitive abilities were included in four studies [14, 41, 43, 46], comprising 32% of the pooled autistic sample (191 autistic children and adolescents). Of studies reporting sufficient information regarding participant characteristics, 44% of the sample (261 autistic children and adolescents) were identified as participants with limited verbal and/or cognitive abilities or as ‘severely affected’. The remaining 24% of study participants (146 autistic children and adolescents) were categorised as not ‘severely affected’ in line with the criteria developed by Stedman et al. [27] (Additional file 3).

**Table 1:**
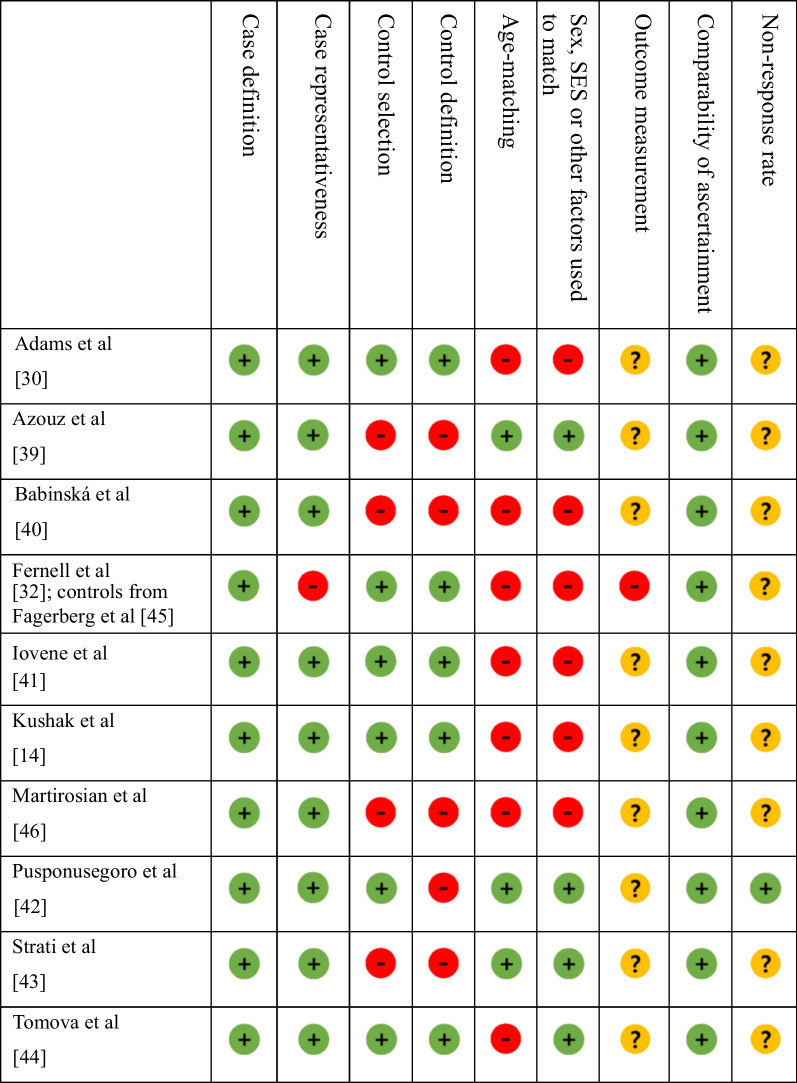
Risk of bias (ROB) of studies of calprotectin and lactoferrin included in the meta-analysis

No risk of bias in line with relevant criteria  Insufficient information to judge risk of bias  Risk of bias in line with relevant criteria

## Discussion

The meta-analyses suggests limited evidence for elevated GI inflammation in autistic children and adolescents relative to non-autistic controls, as indicated by no differences in faecal calprotectin and lactoferrin levels. While a previous systematic review suggested that low-grade GI inflammation might be a major factor in the aetiology of autism [12], our results indicate that this is unlikely at the population level based on the included studies. These findings complement the recommendations of the European Society for Paediatric Gastroenterology and Nutrition Gastroenterology Committee that calprotectin testing is not useful in autistic children in the absence of clinical indications of GI conditions [47]. Many markers of GI inflammation (e.g. M2-PK, S100A12, Alpha1-antitrypsin, etc.) have not been examined in the autistic population. Case-control studies examining rectal nitric oxide [32], faecal lysozyme [30], PMN-E [30], cortisol [31] and glutamate metabolites [31] report low numbers, while conflicting results were reported for faecal IgA levels in autism [30, 33], thus replication is required.

The lack of group differences in faecal calprotectin and lactoferrin levels suggest that GI symptoms in the majority of autistic children and adolescents studied to date and included in this meta-analyses, may not be driven by inflammatory causes. Instead, one explanation is that GI symptoms may be the product of functional GI disorders (FGID) such as irritable bowel syndrome, functional constipation, or functional abdominal pain rather than reflecting the presence of GI inflammation in many autistic children and adolescents [48, 49]. While FGIDs are not associated with changes in markers of GI inflammation [50], there is some preliminary evidence to suggest other immune and GI markers may be associated with FGIDs in the autistic population. FGIDs in autistic participants were associated in a small study with the presence of systemic inflammation as reflected in elevated levels of IL-6 (Interleukin 6) and IFN-γ (interferon gamma) and distinct microbial signatures [51]. Increased rates of GI symptoms such as constipation may alternatively be mediated by the food selectivity associated with autism, rather than organic causes [52, 53].

While there is no evidence in these meta-analyses for elevated GI inflammation at the population level, further exploration of the heterogeneity across studies suggests that there may be a subgroup of autistic children and adolescents who have higher levels of GI inflammation, as reflected in higher calprotectin levels, who may have been missed by underpowered studies. Of the literature reviewed, a few studies extended their investigation to examinations of associations between calprotectin levels, GI symptoms, autistic traits and other biological markers (Table 2).

**Table 2:** Associations reported between calprotectin levels, levels of autistic traits, GI symptoms and other biological markers

*GI symptoms*		
6-Item Gastrointestinal Severity Index (6-GSI) questionnaire [58]	Azouz et al. [39]	A moderate correlation was found between the faecal calprotectin and gastrointestinal symptoms as indicated by parent-reported 6-GSI severity (*r* = 0.471, *p* = 0.002) in a sample of 40 autistic children aged between 3 to 12 years (Mage = 6.53 ± 2.10)
	Laghi et al. [35]	No significant differences were found in the calprotectin levels of autistic preschoolers (average age: 4.14 ± 1.01 years) without GI symptoms (n = 52, median = 79.27 μg/g, IQR = 131.15 μg/g) and those with GI symptoms (n = 28, median = 69.50 μg/g, IQR = 131.21 μg/g)
Full GSI [59], an adaptation of a modified Truelove and Witts Severity Index used for ulcerative colitis clinical trials [60]	Alookaran et al. [38]	No significant differences were found in calprotectin levels in comparisons of controls (*n* = 20, M_age_ = 9.8, SD = 3.8), autistic children and adolescents without GI symptoms (parent-reported GSI score of < 7) (*n* = 10, M_age_ = 8.1, SD = 3.1) and autistic children with GI symptoms (*n* = 20, M_age_ = 9.3, SD = 3.8) aged between 4–16 years
Rome III criteria for constipation [61]	Strati et al. [43]	No difference in calprotectin levels was reported between 5 constipated (based on the Rome III criteria) and 29 non-constipated autistic participants (Mage = 11.1 ± 6.8). No significant differences were also found in comparisons of calprotectin levels between 11 constipated and 29 non-constipated control participants (Mage = 9.2 ± 7.9)
Other GI measures	Tomova et al. [44]	A moderate positive correlation was found between calprotectin levels and parent-reported GI scores based on 5 types of GI symptoms (*r* = 0.36, *p* < 0.05) in a sample of 63 autistic boys aged 2.8–9.2 years (M_age_ = 5.00, SEM = 0.20)
	Bramati-Castellarin et al. [57]	A negative association was found between calprotectin levels and an item in a study specific parent-report measure which asked whether or not their child experienced constipation (*β* = − 1.58, SE = 0.69, *p* = 0.022) in a sample of 49 autistic children (46 male and 3 female) aged between 3.5 and 8 years
*Total level of autistic traits*		
Autism Diagnostic Observation Schedule, Second Edition (ADOS-2) [62]	Tomova et al. [44]	A positive weak correlation was found between ADOS-2 total scores and calprotectin (*r* = 0.29; *p* < 0.05) in a sample of 63 autistic boys aged 2.8-9.2 years (M_age_ = 5.00, SEM = 0.20)
Childhood Autism Rating Scale (CARS) [63]	Azouz et al. [39]	No significant correlation was found between CARS total scores and calprotectin (*r* = 0.175, *p* = 0.280) in 40 autistic children aged between 3 to 12 years (M_age_ = 6.53 ± 2.10). 57.5% of the children were categorised as having mild to moderate scores and 42.5% were categorised as having severe CARS scores
	Iovene et al. [41]	No significant correlation was found in univariate analyses of calprotectin and total CARS scores (*r* = 0.278, *p* = 0.075) in a sample of 47 autistic children (M_age_ = 6.0 ± 2.8) A significant correlation was found between CARS scores and calprotectin levels in a multivariate analysis that adjusted for sex and the presence or absence of GI symptoms, polymorphic leukocytes and specific strains in cultured samples (Lactobacilli, Anaerobic bacteria and Clostridium) (*R*_partial_ = 0.622; *p* = 0.0044)
Approach Withdrawal Problem Composite-subtest of the Pervasive Developmental Disorder Behaviour Inventory (PDDBI) [64]	Pusponegoro et al. [42]	There was no difference found between the median calprotectin in the control group (108.1 µg/g faeces, range 5.3–3595), the group categorised with ‘mild maladaptive’ autism (100.54 µg/g faeces, range: 5.3–3536) and in the group categorised as having ‘severe maladaptive’ autism (56.37 µg/g faeces, range: 5.3–2778) (*p* = 0.80) in a sample of 60 healthy controls, 102 children with ‘mild maladaptive’ autism, and 44 with ‘severe maladaptive’ autism aged between 2 to 10 years
*Social affect and reciprocal social interaction challenges*		
Autism diagnostic interview schedule-revised (ADI-R) Reciprocal social interaction subscale [65]	Babinská et al. [40]	A moderate correlation was found between calprotectin levels and ADI-R reciprocal social interaction subscale (*r* = 0.30, *p* < 0.01) in a sample of 87 autistic children and adolescents aged between 2 and 17 (M_age_ = 7.2 ± 3.8 years)
	Tomova et al. [44]	A weak correlation was found between calprotectin levels and the ADI-R reciprocal social interaction subscale (*r* = 0.26, *p* < 0.05) in a sample of 63 autistic boys aged 2.8–9.2 years (M_age_ = 5.00, SEM = 0.20)
ADI-R communication [65]	Babinská et al. [40]	A moderate correlation was found between calprotectin levels and the ADI-R communication subscale (*r* = 0.38, *p* < 0.001) in a sample of 87 autistic children and adolescents aged between 2 and 17 (M_age_ = 7.2 ± 3.8 years)
ADOS-2 Social Affect subscale [62]	Tomova et al. [44]	A moderate correlation was reported between the social affect subscale of the ADOS-2 and faecal calprotectin (*r* = 0.32, < 0.010) in a sample of 63 autistic boys aged 2.8–9.2 years (M_age_ = 5.00, SEM = 0.20)
ADOS-2 Reciprocal Social interaction subscale [62]	Tomova et al. [44]	A moderate correlation was reported between the reciprocal interaction subscale of the ADOS-2 and faecal calprotectin (*r* = 0.32, < 0.010) in a sample of 63 autistic boys aged 2.8–9.2 years (M_age_ = 5.00, SEM = 0.20)
*Restricted and repetitive behaviours and interests*		
ADI-R restrictive, repetitive behaviours [65]	Babinská et al. [40]	A moderate correlation between calprotectin levels and the ADI-R restricted repetitive behaviours (*r* = 0.33, *p* < 0.01) in a sample of 87 autistic children and adolescents aged between 2 and 17 (M_age_ = 7.2 ± 3.8 years)
Other measures of autistic traits	Bramati-Castellarin et al. [57]	A positive association was found between levels of calprotectin and parent-reported need for fixed routines (*β* = 3.23, SE = 0.81, *p* 0.00009) in a sample of 49 autistic children (46 male and 3 female) aged between 3.5 and 8 years
*Other biological measures*		
Serum markers	Tomova et al. [44]	A moderate positive correlation was identified between *MIP-1β* levels and calprotectin levels (*r* = 0.38, *p* < 0.05) in a sample of 63 autistic boys aged 2.8–9.2 years (M_age_ = 5.00, SEM = 0.20)
	Tomova et al. [66]	A weak positive correlation was identified between *plasma levels of S100B* and faecal calprotectin (*r* = 0.21, *p* < 0.05) in 93 autistic children and adolescents aged between 2 and 16 years (M_age_ = 6.22, SEM = 0.30)
Bacterial populations	Tomova et al. [44]	A strong positive correlation was identified between *Costridiacae populations* and calprotectin (*r* = 0.5; *p* < 0.001), in a sample of 63 autistic boys aged 2.8–9.2 years (M_age_ = 5.00, SEM = 0.20)
	Laghi et al. [35]	The amount of *Akkermansia muciniphila* was found to have a moderate negative correlation with calprotectin levels above 50 μg/g (*r* = − 0.32; *p* = 0.041) in a sample of 80 preschoolers (Age = 4.14 ± 1.01) The amount of *Prevotella* was strongly associated with calprotectin levels above 200 μg/g (*r* = 0.75, *p* = 0.003) The study did not report the proportion of children who had calprotectin levels above 50 μg/g or above 200 μg/g
Fungal populations	Iovene et al. [41]	No significant correlation was found in univariate analysis of calprotectin and levels of Candida (*r* = − 0.019, *p* = 0.90) in a sample of 47 autistic children (Mage = 6.0 ± 2.8) No significant correlation was found in the subsequent multivariate analyses which adjusted for sex and the presence or absence of GI symptoms, polymorphic leukocytes and specific strains in cultured samples (Lactobacilli, Anaerobic bacteria and Clostridium)
Intestinal permeability	Iovene et al. [41]	No significant correlations were found in univariate analyses of calprotectin and intestinal permeability as assessed with the lactulose/mannitol test (*r* = − 0.010, *p* = 0.593) in a sample of 47 autistic children (M_age_ = 6.0 ± 2.8) No significant correlation was found in the subsequent multivariate analysis which adjusted for sex and the presence or absence of GI symptoms, polymorphic leukocytes and specific strains in cultured samples (Lactobacilli, Anaerobic bacteria and Clostridium)

Despite the limitations of the variability in methods used to characterise GI symptoms, the results of the six studies which analysed associations with GI symptoms suggest that the higher levels of GI symptoms may correspond with higher calprotectin levels in some children and adolescents. The median levels of calprotectin in studies where all autistic participants have GI concerns report calprotectin levels above thresholds indicating the presence of inflammation. Recommended cut-offs indicative of the presence of GI inflammation range between 50 and 200 ug/g depending on the need to balance specificity and sensitivity [54, 55]. Kushak et al. found that the mean reported levels among autistic children and adolescents with GI conditions was 111 µg/g, indicative of low levels of GI inflammation and was elevated in 63% of the samples from autistic participants [14]. Azouz et al. [39] reported calprotectin levels above 50 ug/g in 35% of their population and found a significant association between the presence of high levels of GI symptoms and calprotectin levels. De Magistris et al. [36] found elevated calprotectin levels in 24.6% of their participants; however, GI symptom severity was not examined in their study. It is likely however that not all GI symptoms are associated with higher levels of calprotectin, and calprotectin levels did not discriminate between autistic children with GI symptoms and those without in the small studies that have been carried out to date [35, 38, 43]. In assessing GI symptoms in autistic populations, it is important to consider that the ways in which autistic children communicate the presence of GI discomfort will differ based on their verbal ability [3]. Symptoms such as irritability, aggression, obsessive–compulsive behaviours, anxiety, heightened sensory responses and sleep difficulty may indicate the presence of abdominal discomfort in non-verbal children [10, 56].

These atypical manifestations of GI symptoms in the autistic population may also contribute to the associations some studies have found between the presence of GI inflammation and clinical presentations of autism. The results of studies using different measures of autistic traits were conflicting and there was substantial variability in the results of the seven studies which examined associations between faecal calprotectin and autistic traits. Moderate positive correlations were reported between calprotectin levels and level of autistic traits based on the Autism Diagnostic Interview Schedule (ADI-R) and the ADOS-2 [40, 44] and self-reported measures of social behaviour and communication [57]. Associations between calprotectin and autistic traits appear dependent on the categorisation of these traits way severity is defined, with no difference in calprotectin levels between participants characterised as ‘severe’ versus ‘mild’ by the Approach Withdrawal Problem Composite-subtest of the Pervasive Developmental Disorder Behaviour Inventory (PDDBI) [42]. The results of studies using the Childhood Autism Rating Scale (CARS) are conflicting with some studies finding positive correlations between higher calprotectin levels and elevated CARS scores [41] others did not [39].

The question of how to best identify which autistic people have GI inflammation and associated GI conditions remains. This is of particular importance as a recent meta-analysis identified a 1.66-fold increase in IBD rates among children and adults with a diagnosis of autism [13]. In the reverse direction, a population-based study of childhood-onset IBD found an increased hazard ratio of 1.4 in autism diagnoses relative to controls without IBD [67]. This suggests that while there may not be a difference in the levels of GI inflammation at the population level, these case-control comparisons likely mask a subset of autistic people who experience higher levels of GI inflammation and may have higher incidences of associated GI disorders. Calprotectin is commonly used in clinical practice for the identification and monitoring of IBD [19], and the heterogeneity of the differences reported by included calprotectin studies suggest that calprotectin may be able to identify subsets of autistic people with elevated GI inflammation. These individuals may also have specific clinical presentations and immune system abnormalities which warrant further exploration. To enable better identification of these children and adolescents, future studies should recruit adequately powered sample sizes and report what proportion of sampled autistic participants have calprotectin levels above established, age-specific cut-offs. To improve screening and, in turn, quality of life for autistic people with IBD, future studies examining calprotectin should prioritise targeted research designed to identify factors associated with elevated inflammation and IBD diagnoses, rather than examining GI inflammation in the broader autistic population.

## Limitations

There are methodological issues in the published papers included in this systematic review and meta-analyses that limit our understanding of GI inflammation within the autistic population. There was significant heterogeneity in the studies assessing calprotectin which may have been in part due to the limited screening of the non-autistic participants in many studies. Including non-autistic participants with GI issues in control groups confounds the results of a number of studies and limits our capacity to draw conclusions about the relationship between faecal biomarkers of GI inflammation and autism. For example, controls with high levels of calprotectin may have conditions associated with GI inflammation such as coeliac disease, viral gastroenteritis, and or use specific classes of medication such as non-steroidal anti-inflmmatory drugs (NSAIDs) (e.g. Ibuprofen) and proton pump inhibitors [68–71]. It is recommended that future case–control studies carefully screen non-autistic controls for GI issues prior to recruitment and subset autistic participants into those with and without GI symptoms. Studies of GI inflammation should also at a minimum collect detailed data from all participants on the presence of GI symptoms and any diagnoses of GI diseases (e.g. coeliac disease or IBD) and exclude those who had used medications such as NSAIDs, protein pump inhibitors, probiotics or who may have had gastroenteritis in the two weeks preceding sample collection.

As our systematic review and meta-analyses were limited to studies which fulfilled our aforementioned search criteria, it is possible that the subjects included in these meta-analyses may not fully represent the autistic population as a whole. Faecal calprotectin and lactoferrin show good sensitivity (calprotectin: 0.978, lactoferrin: 0.81–0.82) and specificity (calprotectin: 0.682; lactoferrin: 0.71–0.82) [72, 73]; however, a minority of people with active IBD may not show elevated levels of calprotectin or lactoferrin [74, 75]. The majority of autistic participants included in the analysed studies were male. No studies published to date included enough autistic girls to enable an analysis of whether there are sex differences in the prevalence of GI inflammation within the autistic population. Failure to control for age was common among the studies, with only two studies reported appropriate age matching which also compromised the quality of the data included in these meta-analyses. This may particularly affect the validity of studies investigating calprotectin levels in children, as calprotectin levels tend to be physiologically higher in healthy children aged four years of age or younger relative to older children [76–78]. None of the studies included in the analyses reported significant differences in the ages of control and autistic participants; however, given the association of age with higher calprotectin levels, age should be explicitly accounted for. To ensure the accuracy of findings, future studies including children aged four or under should instead 1:1 age-match participants, carry out separate analyses of children aged over and under four years, or account for age as a potential confounder during analysis (e.g. paired tests or appropriate regression models). Studies of GI inflammation should also be carried out in adult populations particularly given elevated genetic risk of GI cancers (colorectal and pancreatic) in autistic populations found in some studies [79, 80].

## Conclusions

Synthesis of the literature published to date provides no evidence of differences in faecal calprotectin and lactoferrin levels between autistic children and non-autistic children however we found signifcant heteogeneity in studies of calprotectin. Future studies should collect detailed clinical and biological data from both autistic and non-autistic participants. This may facilitate a better understanding of which autistic people are at risk of developing inflammation-linked GI conditions rather than exclusively focusing on case–control comparisons as in most studies published to date. Of the studies that considered the heterogeneity of autism, higher levels of calprotectin were associated with a broader range of GI symptoms and higher levels of autistic traits, particularly when measures of social communication differences were examined.

There remain significant gaps in our understanding of how to best identify GI inflammation within the autistic population. Many of the included studies are underpowered, heterogeneity was not well-controlled, and there was limited appropriate screening of control participants for GI issues leading to potential selection bias. Future studies should focus on the generation of high-quality studies with careful screening of non-autistic participants and adequate categorisation and analysis of the clinical and biological heterogeneity of autism, instead of the narrow focus on assessing population level differences in GI inflammation that dominates studies published to date.

### Supplementary Information

**Additional file 1.** Search strategy and study-specific adaptation of the Newcastle-Ottowa Scale for case-control studies.

**Additional file 2.** Additional analyses.

**Additional file 3.** Meta-analysis dataset.

## Data Availability

All data used for the meta-analysis is available in Additional file 3.
