# Supplementary material for: The search for gastrointestinal inflammation in autism: a systematic review and meta-analysis of non-invasive gastrointestinal markers
Source: Mol Autism. 2024 Jan 17;15:4. doi: 10.1186/s13229-023-00575-0 (PMC10795298; doi:10.1186/s13229-023-00575-0)
Supplement: Supplementary file 1 — Additional file 1. Search strategy and study-specific adaptation of the Newcastle-Ottowa Scale for case-control studies. [file 13229_2023_575_MOESM1_ESM.docx]

**Supplementary 1.**

**1.1 PUBMED Search terms**

**Population keywords**

1: Child Development Disorders, Pervasive [MeSH] OR Autism Spectrum Disorder [MeSH] OR Autism Spectrum Disorder [MeSH] OR Asperger Syndrome [MeSH] OR Asperger Syndrome [MeSH] OR Asperger*[All Fields] OR Pervasive Develop* [All Fields] OR Childhood Disintegrative Disorder [All Fields] OR autism [All Fields] OR autist* [All Fields] OR regressive autism [All Fields]

**Biomarker keywords**

**Gut Inflammation:**

2: Feces [MeSH] OR Leukocyte L1 Antigen Complex [MeSH] OR Lactoferrin [MeSH] OR S100A12 Protein [MeSH] OR Pyruvate Kinase [MeSH] OR Neopterin [MeSH] OR Peroxidase [MeSH] OR Leukocyte Elastase [MeSH] OR Lysozyme [MeSH] OR Chitinase-3-Like Protein 1 [MeSH] OR alpha 1-Antitrypsin [MeSH]

3: Stool [All Fields] OR Fecal [All Fields] OR Feces [All Fields] OR calprotectin [All Fields] OR Leukocyte L1 complex [All Fields] OR lactoferrin [All Fields] OR Faecal lactoferrin [All Fields] OR S100A12 [All Fields] OR M2PK [All Fields] OR Neopterin [All Fields] OR Metalloproteinase [All Fields] OR Myeloperoxidase [All Fields] OR Polymorphonuclear elastase [All Fields] OR PMN elastase [All Fields] OR Lysozyme [All Fields] OR alpha 1-Antitrypsin [All Fields]

4: Feces [MeSH] OR Stool [All Fields] OR Fecal [All Fields] OR Feces [All Fields]) AND Cytokine* [All Fields]

5: 3 AND (4 OR 5 OR 6)

**1.2 Risk of bias (quality) assessment**

The quality of case-control studies will be assessed using an adaption of the Newcastle–Ottawa Scale (NOS)

1. Participant selection
a. Case definition
Diagnosis independently confirmed using standardised tools (1)
Diagnosis without standardised tools (0)

b. Representativeness
Inclusion and exclusion criteria appropriate to subgroup (e.g. study of high functioning autism that excludes IQ <70) or broader autistic population and recruited through community sampling or diagnostic clinics (1)
Criteria may limit generalizability (e.g. excluding participants with ID), recruitment from other clinical settings or insufficient detail (0)

c. Control selection
Community recruitment (1)
Recruitment from family members or clinical samples (0)
Potential for selection bias or limited description (0)

d. Control definition
Control status confirmed using standardised assessment, screening tool or people with no family history of autism (1)
Control status poorly defined (0)

2. Comparability of cases and controls
a. Age-matched (1)
b. Sex-matched (1)

3. Outcomes
a. Outcome measurement
Standardized method, citation or adequate description of method and sensitivity limits by with laboratory blinded to case/control status (1)
Insufficient detail provided regarding the measurement of outcomes (0)

b. Method of ascertainment same for cases and controls
Yes (1)
No (0)

c. Non-response rate
Same for both groups (1)
Non-respondents described or rate different and no designation (0)
