# Supplementary material for: The search for gastrointestinal inflammation in autism: a systematic review and meta-analysis of non-invasive gastrointestinal markers
Source: Mol Autism. 2024 Jan 17;15:4. doi: 10.1186/s13229-023-00575-0 (PMC10795298; doi:10.1186/s13229-023-00575-0)

S**upplementary 2: Additional analyses**

**Supplementary Figure 1.** *Meta-analysis of studies of calprotectin levels using standardised mean differences*

**
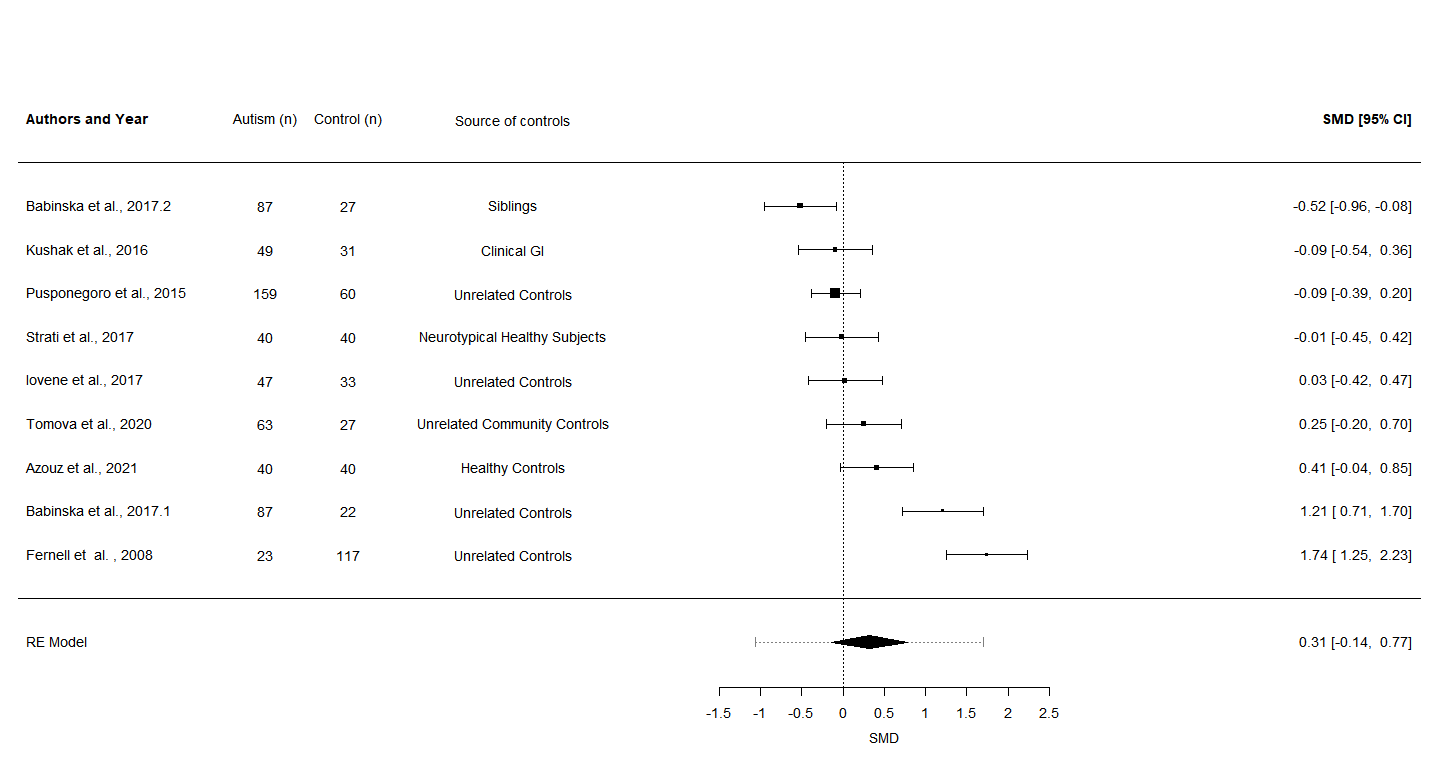
**

**Supplementary Figure 2.** *Meta-analysis of studies of lactoferrin levels using standardised mean differences***
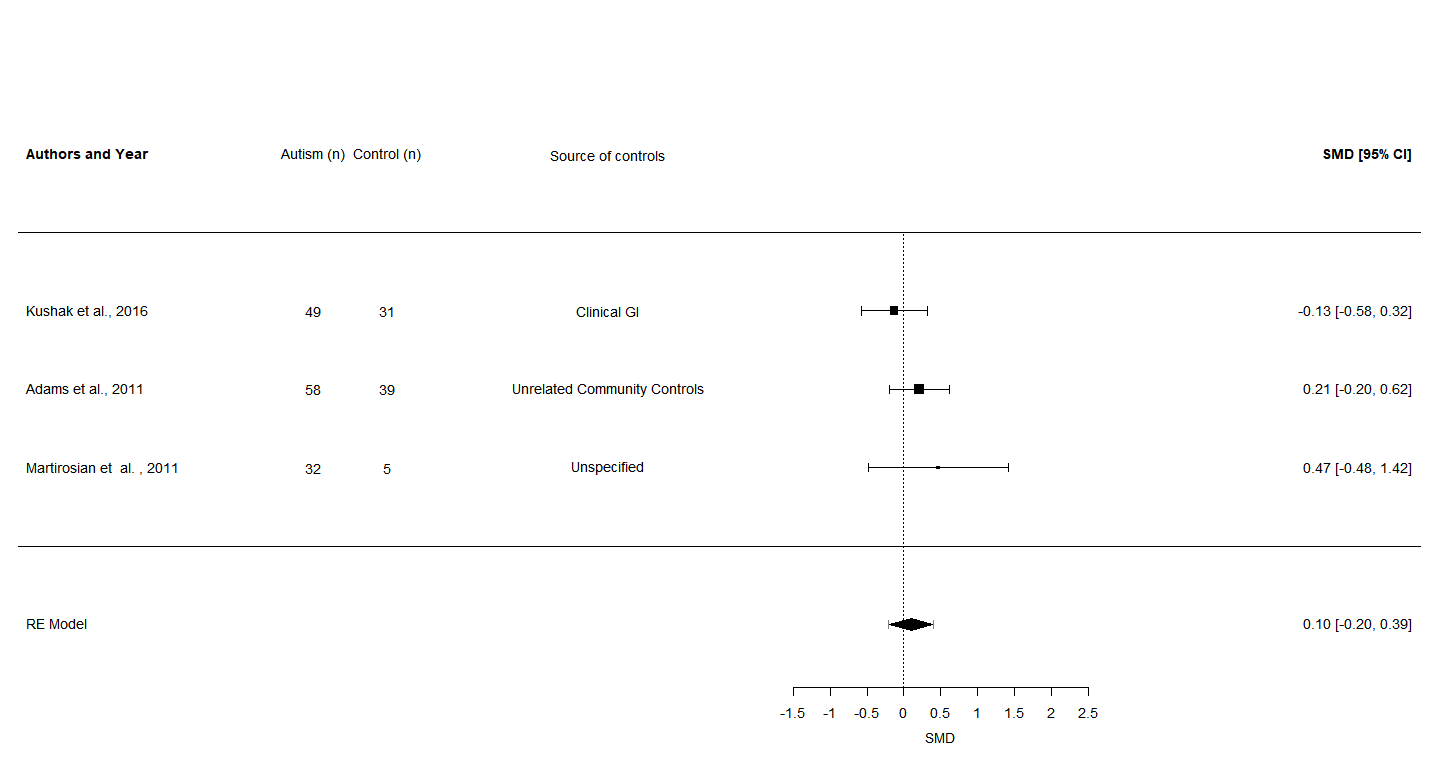
**

**Supplementary Figure 3.** *Meta-analysis of studies of calprotectin levels excluding the Babinská, et al’ (2017)’s sibling comparison group*
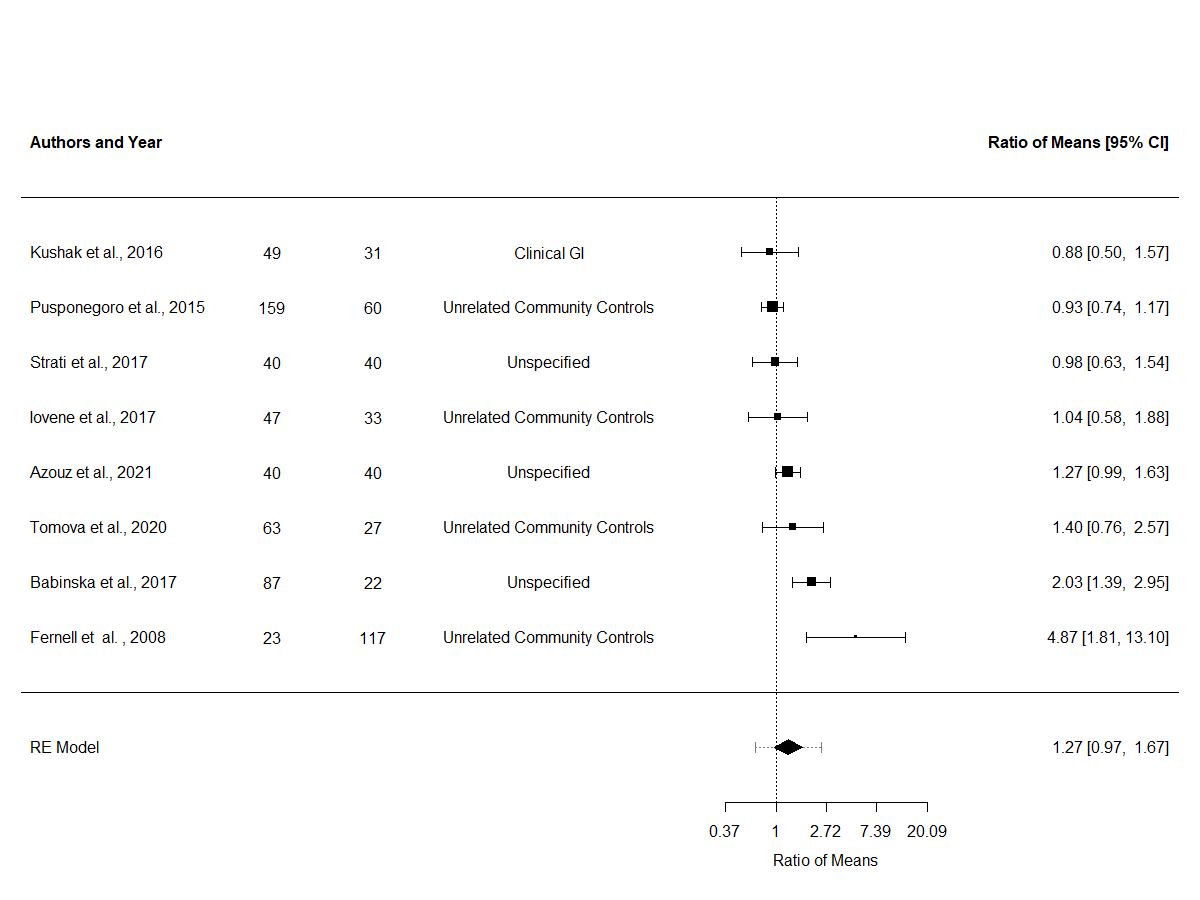

Supplement: Supplementary file 2 — Additional file 2. Additional analyses. [file 13229_2023_575_MOESM2_ESM.docx]
